# A Great Scottish Hospital

**Published:** 1888-09-29

**Authors:** F. Fairbairn Cowan


### A GREAT SCOTTISH HOSPITAL.

EDINBURGH has for centuries been regarded, not only nominally, but actually, as the capital of Scotland—the city in which is stored all the intellectual vigour and scientific life of the country—and as such is looked up to with pride and veneration by the nation at large. The headquarters of scholarship are there, as well as the home of literature and science. "At no period of her history did Edinburgh better deserve her complimentary title of the Modern Athens than the last ten years of the eighteenth and the first ten years of the nineteeneh century," says an English writer. With how much appropriateness this remark can be applied to the Edinburgh of to-day! In Scotland there is no place which can surpass Edinburgh; and it may be questioned if, in regard to its size, there can be found in any country a city which can boast of so many educational and scientific institutions; while the beauty of its situation is unsurpassed, and the excellence of its architecture acknowledged. Among the many noble charitable institutions of which its citizens may justly feel proud, the most conspicuous is the Royal Infirmary—an institution than which there can be none more worthy of regard. It is not a local institution; its benefits are not confined to Edinburgh or its neighbourhood; but its wards receive every year patients from almost every county in Scotland, as well as from England, Ireland, and foreign countries.

It is now nearly a century and a half since the Old Infirmary was built, and during the period of its long existence there has been carried on a work of mercy the greatness of which can never be realised. Its erection was the result of an effort worthy of Scotland and of the generation which then lived, and it benefited the city so greatly that it is still held in the highest estimation by all classes of citizens.

Previous to 1728 the physicians and surgeons of Edinburgh had been in the habit of given gratuitous advice and medicine, but in that year they decided on taking a further step: this was, promoting the erection of a building suitable for the amelioration of their distressed fellow-creatures. In a very short time they had by strenuous efforts collected the then large sum of £2,000. After this was done the contributors were called together by the College of Physicians, and on February 19th, 1728, the first meeting was held, from which date, it may be stated, the minutes of the affairs of the Infirmary have been carefully taken and preserved. At that meeting a report of the subscriptions which had been intimated was read, and a committee was formed for the purpose of collecting them, and also for raising additional subscriptions. Besides, this committee was entrusted with the preparation of a plan for the prospective infirmary. The first general meeting of contributors was held in the same year, on December 16th, when a committee was appointed "to prepare a small house, and get proper servants and the necessaries required to provide for *some* sick." At this meeting there was displayed on the part of the committee of contributors and managers that wisdom and prudence which have always characterised their behaviour in conducting the affairs of this noble institution, and which has again and again brought to light their disinterested zeal for its welfare. It was resolved that the expenditure should only be "so far as the current interest on the stock would go." A small house was soon prepared, and the furnishing was proceeded with as quickly as possible; every precaution being taken to keep down all unnecessary expenditure, but sufficient care being manifested for the comfort of the future patients. Having arrived at this stage of the proceedings, the managers next deemed it advisable to draw up rules and regulations for the administration of their newly-acquired Infirmary. After some deliberation, it was resolved that the number of patients under treatment should not exceed six, and that two women would be sufficient to attend upon them constantly. It may be remarked here that this embryo infirmary was situated near the College, at the head of Robertson's Close. A lease of a house was taken for nineteen years, the rent being fixed at fifty pounds Scots. Things went on so prosperously that August 6th, 1729, witnessed the opening of the "house for receiving sick poor." During the first year thirty-seven patients were treated; the expenditure amounted to £97 19*s.* 7*d.*; and the total capital of the institution was £2,129 19*s.* 7*d.* The sympathy of the public was thoroughly roused by this time, though money did not flow in so readily as the managers desired and expected. The latter found themselves in a dilemma, out of which they extricated themselves by a happy thought. They determined to obtain a charter of incorporation. After many deliberations a petition was presented to his Majesty George II., and, after due consideration, the King was pleased, on August 25th, 1736, to grant a Royal Charter incorporating the contributors. On the receipt of this charter the managers and friends of the small hired house were revived and stimulated; and, finding at the same time that it was impossible to meet the demands made upon them with their limited accommodation, they resolved to build "such an house as can conveniently lodge *all the sick poor who may be supposed to apply for admittance.*" This resolution had the effect of awakening a new interest in the Infirmary; active steps were at once taken, and the result was that on August 21st, 1738, the foundation of the first wing of the buildings in Infirmary Street was laid.

Money now flowed in for the buildings in a way that took the community by surprise, and tradesmen who had little money to give helped on the work by the labour of their hands. The Assembly of the Church of Scotland ordered collections for this purpose at every church door. Societies of different denominations and objects in and around Edinburgh, as well as in other parts of Scotland, helped on the cause, not only by the interest they displayed in its welfare, but by their pecuniary support. No sooner had the first part been completed in 1741 than the managers resolved that "the sick shall be admitted as patients from whatever country or nation they shall come," and it is satisfactory to note this resolution has never been rescinded or annulled. It may not be out of place here to mention a remarkable coincidence in connexion with the date of the opening of the Infirmary. It has already been observed that the foundation-stone was laid in 1738, while the year 1741 witnessed the reception of suffering humanity under its benevolent roof. What is remarkable is that within the same period George Watson's Merchant Academy was commenced and completed on the site on which the present New Royal Infirmary stands.

The action of the managers in admitting distressed sick from any country into the hospital doubled the interest it had awakened throughout Scotland. For many years the managers were enabled to go on smoothly with their beneficent work, and to greatly extend its influence, until at last it was found that the building of 1738 was utterly inadequate to meet the demand upon its resources. Additions, it is true, had been made to the original building as opportunity afforded, but eventually it was found too small, and the necessity of largely increasing the accommodation became only too apparent. Besides, with advanced knowledge, it was felt that the old hospital was ill adapted for the requirements of modern science, and at last it was resolved that a new building should be erected on the site. A committee was appointed to raise money for this purpose. They held a number of public meetings in Edinburgh and other places in Scotland, where the needs and circumstances were stated, and assistance solicited, the result being the awakening of a deep and widespread interest in the work. The response to the appeal was worthy of the object for which it was made, and a subscription was raised greater than had ever been obtained in Edinburgh for any benevolent object. It was in consequence of the great success that the original plan was abandoned of rebuilding on the old spot, and, after many discussions, consultations, and suggestions, one of the finest sites in the city (the building and grounds of George Watson's Hospital) was finally secured, plans were prepared, and on October 13th, 1869, his Royal Highness the Prince of Wales laid the foundation-stone of the New Infirmary. For ten years the preparations and building went on, till at last the magnificent pile was opened on October 29th, 1879, with a ceremonial none the less befitting such an inauguration because it was simple and unostentatious.

The situation of the Infirmary is admirable on account of its proximity to the open spaces of the meadows, Bruntsfield Links, and George Heriot's Hospital; and as the new seat for University medical teaching is close at hand, it also meets the professors' requirements. The architect was guided by the results of medical experience, and what is known as the pavilion system has been followed. This system procures a certain amount of isolation, and is the means of freely circulating air among the various blocks or portions of the whole edifice. The architectural style of the building is that of the old Scottish Baronial of the James V. period, the chief features of which are reproduced in the main frontage in Lauriston. The edifice looks well from any point of view, its beauty being greatly enhanced by its circular turrets, modelled after those of Falkland and Holyrood Palaces.

Each of the separate blocks or pavilions, besides their attics and basements, have three floors, each floor constituting a ward, or separate and independent hospital, capable, if necessary, of complete isolation. The floors are connected by a spacious staircase, and each floor opens out from a wide corridor, at right angles to its upper end. A hydraulic hoist, large enough to hold a bed for the conveyance up or down of a helpless patient, runs from the basement to the top of the block, and there are also shoots for soiled linen, etc., at hand. In short, everything appears to have been considered, and no comfort or convenience seems to have been forgotten; even a complete set of fire-extinguishing apparatus will be found at hand. As a precaution against the germs of disease, the walls are cemented with a mixture containing a disinfectant, while the floors are all of well-varnished Baltic pine. The operating theatres have been constructed with a view to the comfort of students and patients, the principal one, capable of holding about 500 students, having retiring rooms in it, one being specially set apart for the administration of chloroform. A portion of Watson's Hospital is occupied by the general kitchen and nurses' dining hall, and the rooms of the lady superintendent and assistants. In this portion of the building are the dining-room, library, and private apartments of the resident medical staff.

Not the least important department is the dispensing laboratory, situated for convenience between the medical and surgical hospitals. This department is at all times filled with a supply of the best and purest drugs, and is in the charge of a thoroughly competent chemist. For the safety of the patients, the chief or one of his assistants must always be in attendance to meet any emergency.

Besides supplying the temporal wants of the patients, the managers have not forgotten their spiritual requirements. For this purpose there is a neat little chapel, fitted up with a splendid organ, a gift by former nurses. Devotional exercises are conducted on Sunday mornings by "fourth year" students, and in the evenings by the chaplain or some Edinburgh minister.

The pathological department is situated in the north-west corner of the grounds, and apart from the general edifice. Although the architectural features of this isolated block are not so prominent as the rest of the building, yet they harmonise with the general design. The principal feature of this block is a large theatre for lectures. It is seated for 220 students, and has microscopic and chemistry rooms attached. Near it is the mortuary, the walls of which are lined with white glazed bricks. There is a direct communication between it and the surgical and medical hospitals by means of an underground passage through which the bodies of the dead can be conveyed, unseen by the other patients.

In the immediate neighbourhood are the workshops, engine-house, and laundry. The soiled linen is conveyed to the latter through a tunnel, and operated upon by a washer worked by steam, a mechanical wringer, and a hot drying-chamber. Beside the laundry is the engine-house for working the heating apparatus throughout the building, and the hydraulic machinery of the hoists.

A description of one ward may answer all. Coming from out the rush and noise of the streets, a sense of peace and rest is at once felt; and the reflection arises that it may be a good thing for many a man or woman to be obliged to stay for a while in such an atmosphere of perfect cleanliness and order. From hunger, and want, and over-crowded homes, many a patient has been brought to the wards, and, when discharged, has carried away with him lessons which will never be forgotten. Looking down the line of beds, where many cheerful faces can be seen, it is difficult to believe that there the grim struggle against disease and death is always carried on. Day and night there is a continual movement, though never any noise; and when the town is hushed in sleep, there appear lights in the windows of the great building, indicating that the comfort and safety of its poor lodgers are being continually cared for. On entering a ward, the effect is one of brightness and cleanliness, and space; one is immediately struck by the contrast between the spotlessness of nurses, patients, and furniture, and the general dirt which is frequently found in the ordinary private houses of the poorer classes. Each patient is provided with a small table, chair, and a locker for his clothes. At the head of the room a large fire is always kept burning, round which convalescent patients beguile the time by anecdotes of home, travel, and adventure. In the centre of the room a table is placed, usually covered with flowers, the gifts of visitors and patients. After spending a short time in visiting one of these wards, it is almost with a feeling of regret that one takes leave of the place to mix in the throng and noise of a crowded thoroughfare.

The monotony of the patient's life is happily broken by the visits of the "ward visitors"—a society of about ninety ladies—who, by their conversation, sympathy, and other ministries of kindness, add much to the comfort and benefit of the patients. The latter are also deeply indebted to the Flower Mission, and to the medical students, whose brief services in the wards on Sunday mornings are much appreciated. The Samaritan Society also does excellent work, not only helping, as far as its means permit, those families whose bread-winners are laid aside by sickness, but also assisting with money and clothing the more necessitous among the patients on their leaving the institution.

The relations between the University and the Infirmary have at all times been mutually beneficial. The Medical School of the University has always followed the fortunes of the Infirmary, and, as stated, is now quartered side by side with it. Wards have been set aside for the professors of medicine and surgery, in which they give practical instruction to their students. The daily life of the latter is a subject from which much can be gleaned, and which would form a very interesting chapter.

In concluding, it may not be amiss to remark on the financial condition of the Infirmary. The magnitude of the institution may be understood when it is stated that for site, building, and furnishing a sum of between £340,000 and £350,000 was required. The one great fact that out of 8,823 patients treated last year, 4,037 were dismissed cured, and 2,911 relieved, shows the nature and extent of the very important benefits conferred on the sick and hurt, and indirectly on their relatives and friends, by the eminent skill of the medical and surgical gentlemen in attendance, and the kindly sympathy, attention, and services of the large staff of nurses, etc. In addition to these indoor patients, not less than 200 people have on an average attended daily (Sunday excepted) in the waiting rooms of the different departments—medical and surgical—obtaining the benefit of the high professional skill of the physicians and surgeons, and receiving all necessary dressings and appliances from the institution. The greatly increased facilities now offered in the new buildings for ministering to the suffering are being taken advantage of to a remarkable extent—oftentimes, indeed, the demands cannot altogether be met by the managers. There thus arises a very urgent necessity for increased subscriptions, so that the many patients seeking admission may have that attention paid to them which the managers are most desirous they should receive. It is to be hoped the friends of the institution will continue their support. It is indeed a great and noble work which is carried on in the Infirmary, and it would be a pity, if when called on, the managers were unable, through want of funds, to avail themselves to the fullest extent possible of the benefits the building is capable of affording. It may be added that the total number of beds available in the Infirmary is 670, in addition to thirty cots for children.

F. Fairbairn Cowan.

